# Treatment planning optimization with beam motion modeling for dynamic arc delivery of SBRT using Cyberknife with multileaf collimation

**DOI:** 10.1002/mp.13848

**Published:** 2019-10-22

**Authors:** James L. Bedford, Henry S. Tsang, Simeon Nill, Uwe Oelfke

**Affiliations:** ^1^ Joint Department of Physics The Institute of Cancer Research and The Royal Marsden NHS Foundation Trust London SM2 5PT UK

**Keywords:** arc therapy, noncoplanar trajectory, SABR, SBRT, VMAT

## Abstract

**Purpose:**

The use of dynamic arcs for delivery of stereotactic body radiation therapy (SBRT) on Cyberknife is investigated, with a view to improving treatment times. This study investigates the required modeling of robot and multileaf collimator (MLC) motion between control points in the trajectory and then uses this to develop an optimization method for treatment planning of a dynamic arc with Cyberknife. The resulting plans are compared in terms of dose‐volume histograms and estimated treatment times with those produced by a conventional beam arrangement.

**Methods:**

Five SBRT patient cases (prostate A — conventional, prostate B — brachytherapy‐type, lung, liver, and partial left breast) were retrospectively studied. A suitable arc trajectory with control points spaced at 5° was proposed and treatment plans were produced for typical clinical protocols. The optimization consisted of a fluence optimization, segmentation, and direct aperture optimization using a gradient descent method. Dose delivered by the moving MLC was either taken to be the dose delivered discretely at the control points or modeled using effective fluence delivered between control points. The accuracy of calculated dose was assessed by recalculating after optimization using five interpolated beams and 100 interpolated apertures between each optimization control point. The resulting plans were compared using dose‐volume histograms and estimated treatment times with those for a conventional Cyberknife beam arrangement.

**Results:**

If optimization is performed based on discrete doses delivered at the arc control points, large differences of up to 40% of the prescribed dose are seen when recalculating with interpolation. When the effective fluence between control points is taken into account during optimization, dosimetric differences are <2% for most structures when the plans are recalculated using intermediate nodes, but there are differences of up to 15% peripherally. Treatment plan quality is comparable between the arc trajectory and conventional body path. All plans meet the relevant clinical goals, with the exception of specific structures which overlap with the planning target volume. Median estimated treatment time is 355 s (range 235–672 s) for arc delivery and 675 s (range 554–1025 s) for conventional delivery.

**Conclusions:**

The method of using effective fluence to model MLC motion between control points is sufficiently accurate to provide for accurate inverse planning of dynamic arcs with Cyberknife. The proposed arcing method produces treatment plans with comparable quality to the body path, with reduced estimated treatment delivery time.

## 1 Introduction

Cyberknife is a well‐established device for delivering high‐quality dose distributions in radiotherapy.^1^, ^2^ It consists of a short‐waveguide 6‐MV flattening‐filter‐free linear accelerator mounted on a robotic arm. Collimation is by means of a series of circular collimators, a variable circular diaphragm, or a multileaf collimator (MLC).^1^ The MLC allows for faster delivery of treatments to larger tumors with fewer monitor units. The device is particularly well‐suited to treatment of stereotactic body radiotherapy (SBRT), where its ability to adopt a variety of noncoplanar beam orientations and to shape the radiation beam intricately allows a focused dose of radiation to be delivered.

The Cyberknife typically traverses through up to 100 beam positions during delivery of a fraction of radiotherapy.^3^ This provides for a very conformal dose distribution but usually takes a long time to deliver. In the last decade, delivery time has been considerably reduced on conventional C‐arm linear accelerators by the introduction of volumetric modulated arc therapy (VMAT), wherein the gantry of the accelerator is moved through a range of positions with the treatment beam continuously on.^4^, ^5^, ^6^ Several authors have investigated the application of this type of approach to Cyberknife. Kearney et al.^7^ describe a noncoplanar arc optimization algorithm for Cyberknife with a circular collimator. Their method uses a four‐step approach which determines orientations, beams and collimator sizes, calculates source trajectories, generates intermediate radiation models, and finally calculates monitor units. A further study provides an arc optimization algorithm for the Cyberknife with MLC, which includes a direct aperture optimization step after determination of the beam trajectory.^8^

Accurate computation of the dose delivered by such arcing techniques requires that the continuous delivery be modeled accurately. Kearney et al.^8^ achieve this through the use of dense sampling of intermediate apertures between the key beams used to define the trajectory. In the context of SBRT, this step is important as the MLC moves a large distance in relation to the size of the aperture. Recently, Christiansen et al.^9^ have reported on an efficient method for performing such a sampling in the context of VMAT delivery. By the use of ramp functions to model the fluence delivered between control points, accurate dose calculation and optimization can be achieved without the need to optimize with a very fine control point spacing or perform time‐consuming aperture interpolation.

This study investigates the performance of arc delivery using the Cyberknife with MLC, for the case of SBRT. Performance is measured in terms of dose‐volume histograms, clinical dose‐volume constraints, and estimated treatment times. An optimization method is described, and then applied to several clinical cases. To overcome the risk of collision, which is always present when choosing a noncoplanar arc trajectory,^10^, ^11^, ^12^, ^13^, ^14^, ^15^, ^16^ a fixed trajectory is used. Plans are optimized using the continuous aperture calculation method^9^ to model the motion of the MLC between control points. The accuracy of this approach is evaluated by explicitly comparing against plans with interpolated beams. The quality of treatment plans in terms of dose‐volume histograms, conformity indices, calculated monitor units, and expected delivery times are evaluated against the corresponding plans using a fixed body path.

## 2 Materials And Methods

### 2.1 Optimization scheme

For all cases, the optimization scheme was a three‐step method which optimized a fluence distribution for each beam direction, sequenced the fluence distribution into deliverable apertures, and then performed direct aperture optimization.^15^, ^17^ This method was used for both dynamic arc and step‐and‐shoot plans, the difference between the two types of plan occurring in the sequencing and in the application of delivery constraints during the direct aperture optimization. The exact differences are described later in Section 2.A. The resolution of the fluence map was 7.7 mm × 5 mm at a nominal source‐axis distance of 800 mm. The choice of 7.7 mm was equal to two leaf widths, so that MLC leaves could be paired during sequencing. Dose was calculated as:

(1) $$D_{i}=\sum_{j}d_{\mathrm{ij}}w_{j},$$

where *D_i_* was the dose at voxel *i* in the patient model, *d_ij_* was the dose delivered by a unit fluence at beamlet *j* to voxel *i*, and *w_j_* was the beamlet weight. Fluence was optimized by minimizing an objective function, *F*:

(2) $$F=\sum_{i}f_{i},$$

where the indices, *i*, referred to individual anatomical structures, each with objective value *f_i_*:

(3) $$f_{i}=a_{i}{\left[d_{i}^{\min}-d_{i}\right]}_{\geq 0}^{2}+b_{i}{\left[d_{i}-d_{i}^{\max}\right]}_{\geq 0}^{2}.$$

Both the minimum and maximum terms were used for the planning target volume, while only the maximum term was used for normal tissues. The variables *a_i_* and *b_i_* referred to the importance factors for structure *i*. A gradient descent method was then used to modify the beamlet weights, *w_j_*, so as to minimize the objective function:

(4) $$w_{{}_{j}}^{x+1}={\left[w_{j}^{x}-\alpha p_{j}^{x}\right]}_{\geq 0}.$$

where the superscript *x* denoted the iteration number and α was a relaxation parameter. The direction vector *p^x^* was in principle given as:

(5) $$p^{x}={\left[\nabla^{2}F\left(w^{x}\right)\right]}^{-1}\nabla F\left(w^{x}\right),$$

but as the inverse Hessian matrix ${\left[\nabla^{2}F\left(w^{x}\right)\right]}^{-1}$ was large and therefore memory‐intensive, the low‐memory Broyden–Fletcher–Goldfarb–Shanno (L‐BFGS) method was used to avoid having to explicitly calculate it. The L‐BFGS used a recursion relation^17^ to calculate the direction vectors:

(6) $$p^{x+1}=p^{x}+B\left(F,\nabla F\right),$$

where $B\left(F,\nabla F\right)$ was a direction updating function. Forty iterations of fluence optimization were used in all cases. This number of iterations was chosen empirically to give a moderately well‐optimized plan without introducing a high degree of structure into the fluence maps, which then could not be reproduced during the sequencing step. This was particularly important for the dynamic arc plans, where the number of apertures allowed at the sequencing step was very limited.

Following fluence optimization, sequencing was carried out using the well‐established method of Xia and Verhey.^18^ In the case of arc plans, fluence optimization was performed at every third beam orientation (i.e., with 15° node separation). The resulting fluence maps were sequenced into three apertures and the two additional apertures were redistributed to the beam orientations either side of the fluence map. In the case of step‐and‐shoot plans, all beams were sequenced, with a limit on the maximum number of apertures per plan. The same L‐BFGS method that was used for fluence optimization was then used for direct aperture optimization, with the aperture optimization problem converted into an optimization of effective fluence.^15^ The fluence assigned to a fluence bixel partially covered by an MLC leaf was weighted according to the proportion of the bixel that was exposed. In other words, if the position of an MLC leaf during direct aperture optimization was halfway across a fluence bixel, that bixel was assigned a value of half of the open‐field fluence.

At each iteration of the direct aperture optimization, the MLC and arc delivery constraints were applied. These were as shown in Table 1. With regard to the arc speed parameters, the rationale was to use the robot speed as the key factor. From this, the time to traverse between nodes spaced at 5° was calculated as 1.5 s. The maximum MLC speed of 33 mm/s was suggested by the vendor as a speed that could be achievable with the current MLC design. Using this speed, the allowed motion of the MLC between control points was calculated as 50 mm, and this was used in the optimization.

**Table 1:** Multileaf collimator (MLC) and arc motion constraints used for the study.

Constraint	Value	Comments
Min. field width	7.6 mm	Virtual constraint to ensure sufficiently large open aperture area
Min. field length	7.7 mm	Two leaf pairs
Min. distance to opposing leaf in next leaf pair (i.e., interdigitation situation)	5.0 mm	If distance to opposing leaf in next leaf pair is <5 mm and >0 mm, open leaf to 5 mm. If distance is <0 mm, that is, interdigitating, close leaf pair completely
Max. robot speed	60 mm/s	Comparable to slowest speed on current machine
Min. time to traverse 5° of arc	1.5 s	Calculated from the robot speed constraint
Max. MLC speed	33 mm/s	Faster than current machine configuration but achievable with the current MLC design
Max. leaf motion per 5° of arc	50 mm	Calculated from the MLC speed and traversal time for 5° of arc
Min. monitor units per segment	0 MU	Assuming that the machine can turn dose rate off completely if necessary
Max. monitor units per segment	Not constrained	Assuming that the robot speed can be reduced to deliver higher doses as needed

### 2.2 Calculation of fluence and dose during optimization

The dose influence matrix *d_ij_* was calculated using an Accuray‐supplied pencil‐beam algorithm. A series of bixel‐sized fields were set and the dose calculation used to calculate dose throughout the entire patient volume. The dose grid was 2 × CT pixel size in the transaxial direction and CT slice spacing in the longitudinal direction. Dose voxels which received <0.015% of the maximum dose of each *d_ij_* component were neglected so as to minimize the size of the dose matrices. The dose influence matrix for each beam of the plan therefore required approximately 1 GB. All doses in the study were then calculated as summations of these *d_ij_* component doses.

**Figure 1:**
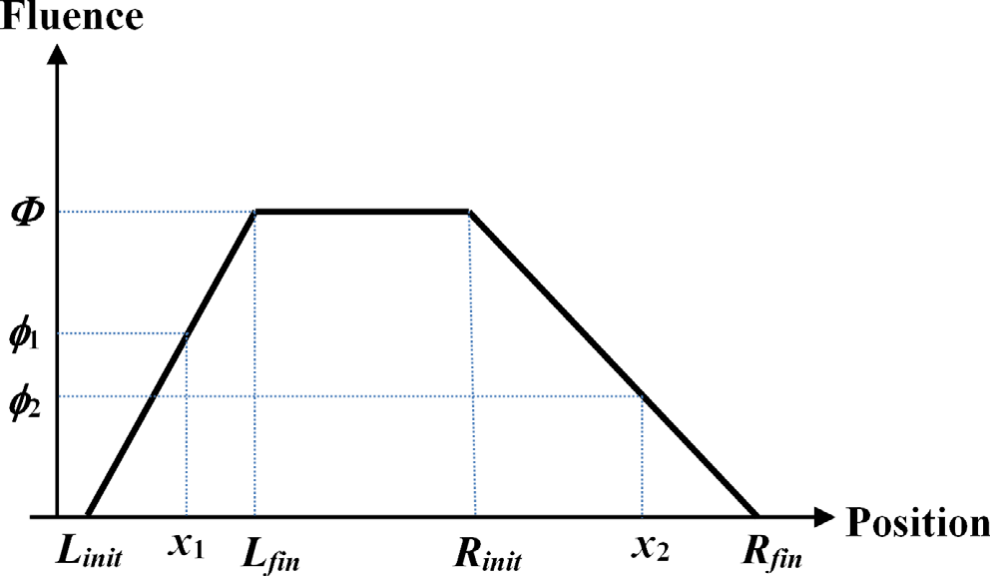
Model of fluence during multileaf collimator leaf motion. The left leaf moves from *L_init_* to *L_fin_* and the right leaf moves from *R_init_* to *R_fin_*. The effective fluence is *ϕ*_1_ at *x*_1_ and *ϕ*_2_ at *x*_2_. [Color figure can be viewed at http://www.wileyonlinelibrary.com]

No modeling of arc motion was carried out during fluence optimization. After sequencing, the allowed MLC motion was included in the optimization, but for dose calculation, one of two methods was used: (a) no motion modeling in the dose calculation, and (b) use of an effective fluence method to model the MLC leaf motion.^9^ The former simply assumed a uniform fluence over the aperture at each control point, with no account taken of the change in the aperture between control points. The latter used the following principles, as illustrated in Fig. 1.

Assuming constant speed, with no acceleration or deceleration, the fluence received by a bixel positioned at *x*_1_ was given by the linear interpolation:

(7) $$\phi_{1}=\Phi \left(\frac{x_{1}-L_{\mathrm{init}}}{L_{\mathrm{fin}}-L_{\mathrm{init}}}\right),$$

where *Φ* was the fluence delivered by the open aperture. The fluence for a bixel positioned at *x*_2_ was given by:

(8) $$\phi_{2}=\Phi -\Phi \left(\frac{x_{2}-R_{\mathrm{init}}}{R_{\mathrm{fin}}-R_{\mathrm{init}}}\right).$$

If the leaves underwent significant motion relative to the aperture width, it was possible for a bixel to lie in both regions simultaneously, that is the ramp down began before the ramp up finished. In this case, the fluence received by the bixel was^9^:

(9) $$\phi =\Phi \left(\frac{x-L_{\mathrm{init}}}{L_{\mathrm{fin}}-L_{\mathrm{init}}}-\frac{x-R_{\mathrm{init}}}{R_{\mathrm{fin}}-R_{\mathrm{init}}}\right).$$

### 2.3 Postoptimization recalculation of dose

**Figure 2:**
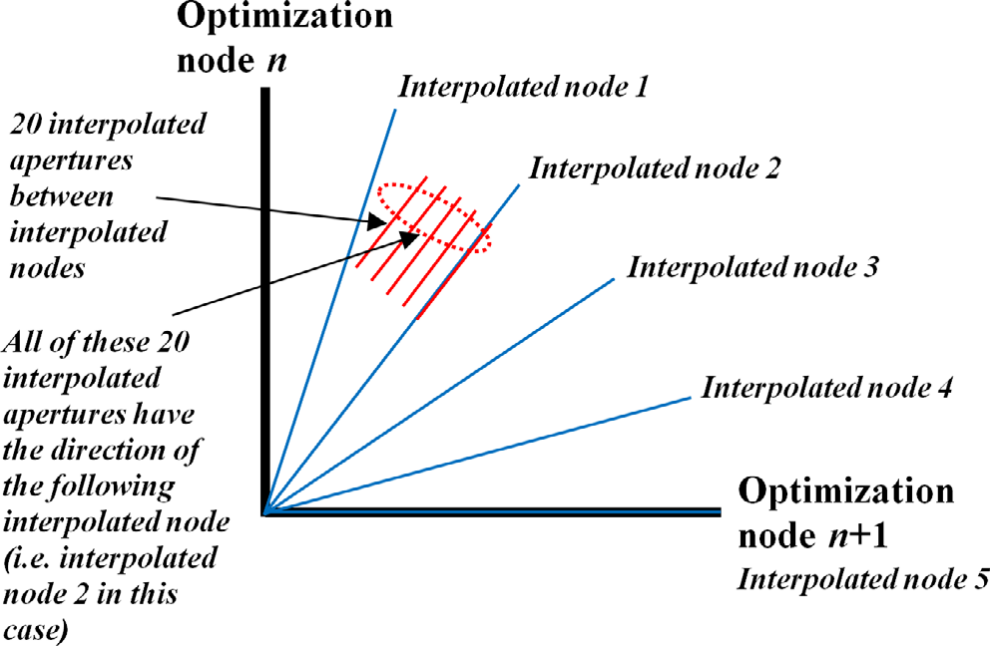
Model of node interpolation during postoptimization recalculation of dose. For each optimized node, there are five intermediate node orientations and 100 interpolated apertures. [Color figure can be viewed at http://www.wileyonlinelibrary.com]

After optimization, the accuracy of the final dose calculation was assessed by adding intermediate interpolated nodes. The change in node orientation between two nodes was modeled by four intermediate nodes, which, together with the second of the two original nodes, formed a set of five interpolated nodes. In some cases, an additional 20 interpolated apertures were added between each of these interpolated nodes, but their directions were coalesced onto the following interpolated node in the manner described by Bedford.^19^ This procedure was to allow the effect of the MLC motion to be included in the dose calculation while restricting the computations to the interpolated nodes in the interests of limiting the time required (Fig. 2). The monitor units were divided equally between the interpolated apertures. At the first node in the nodeset, all the interpolated nodes and apertures were produced, but the shapes were just copies of the first shape as there was nowhere to interpolate to.

**Figure 3:**
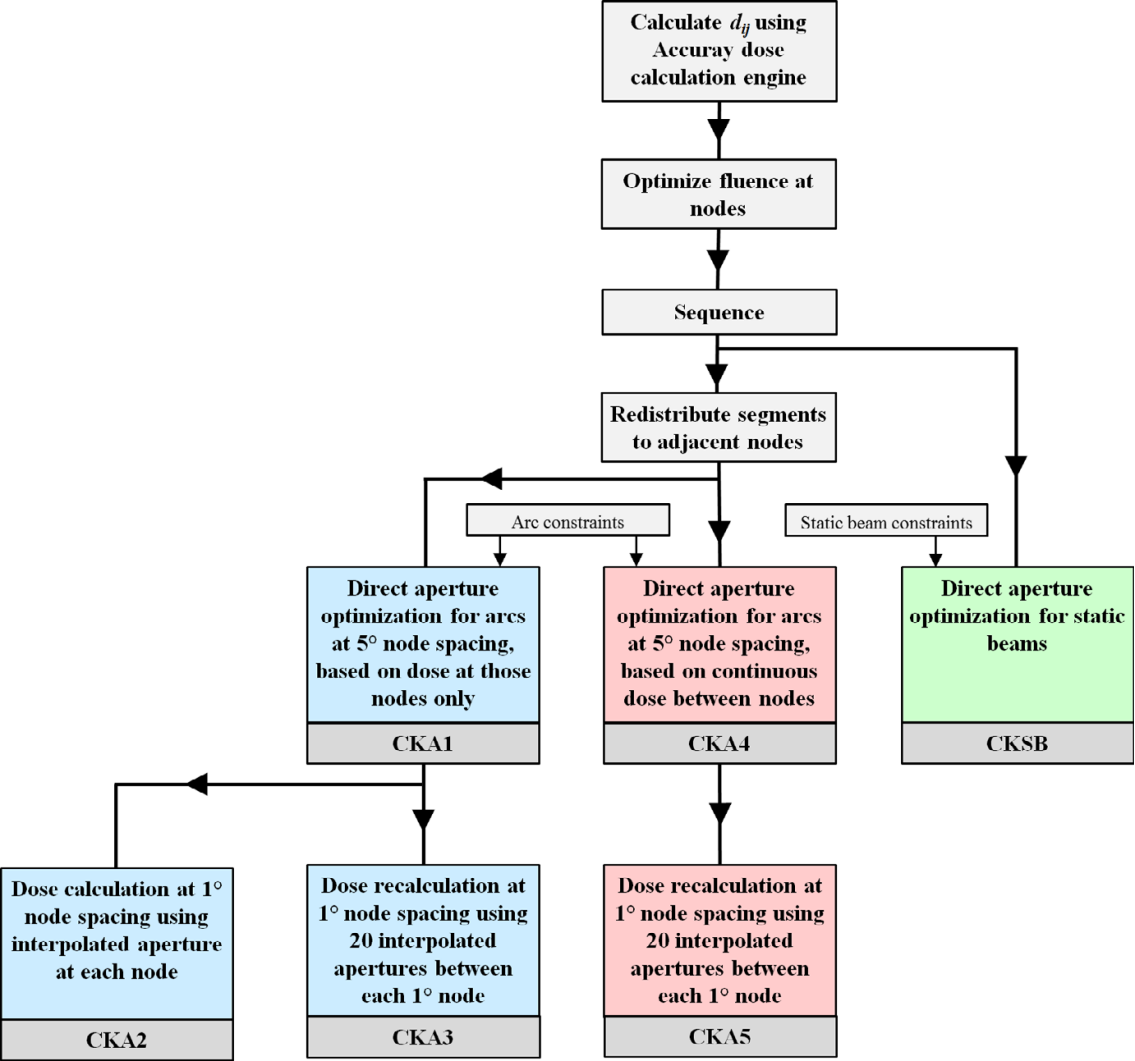
Workflow used for optimization and recalculation of treatment plans. [Color figure can be viewed at http://www.wileyonlinelibrary.com]

The complete workflow used for optimization and recalculation of the treatment plans is shown in Fig. 3. A summary of the SBRT comparisons carried out in this paper is given in Table 2. The plans are for a dynamic intensity‐modulated Cyberknife Arc (CKA) or multiple step‐and‐shoot Cyberknife beams with static beam orientations (CKSB). Methods CKA1, CKA2, and CKA3 are compared in one comparison, methods CKA4 and CKA5 are compared in a separate comparison as they are based on a different dose calculation during optimization and therefore result in a separate plan. Finally, method CKA4 is compared with CKSB.

**Table 2:** Summary of plans and dose calculation methods compared in this paper.

Scheme	Optimization dose calculation		Final dose calculation	
	Description	Effective angular resolution	Description	Effective angular resolution
CKA1	At control points only	5°	Optimization only. No recalculation	5°
CKA2	At control points only	5°	Using five interpolated nodes between each pair of control points	1°
CKA3	At control points only	5°	Using five interpolated nodes between each pair of control points and 20 interpolated apertures between these nodes.^19^	0.05°
CKA4	At control points only, with influence of apertures between control points included.^9^	Continuous	Optimization only. No recalculation	Continuous
CKA5	At control points only, with influence of apertures between control points included.^9^	Continuous	Using five interpolated nodes between each pair of control points and 20 interpolated apertures between these nodes.^19^	0.05°
CKSB	Per beam	Per beam	Optimization only. No recalculation	Per beam

### 2.4 Illustration of motion between control points

The differences between the recalculation strategies were illustrated using a CT scan of a water‐equivalent phantom. The phantom was 300 mm wide by 300 mm long by 200 mm high. A spherical planning target volume (PTV) of approximately 60 mm diameter was located centrally within the phantom and a beam of 800 mm source‐to‐axis distance was directed to a target point situated at the center of the PTV. The beam consisted of just two control points, the first with the beam directed vertically downwards and the second with the beam directed 5° away from vertical (i.e., gantry angle 0° and gantry angle 5° using the IEC 61217 convention). The aperture for the first control point was semicircular and covered half of the PTV, while the aperture for the second control point was also semicircular and covered the other half of the PTV. About 1000 monitor units were assigned to each control point. This plan represented CKA1, and interpolated nodes at 1° intervals were then introduced to represent CKA2. The plan CKA3 additionally included 20 interpolated apertures between the 1° control points.

A further plan was created, in which the aperture of the first control point consisted of 10‐mm MLC leaf openings around the one side of the PTV in a crescent shape, and the aperture of the second control point consisted of similar 10‐mm MLC leaf openings around the other side of the PTV. The first control point had a weight of 500 MU, while the second control point had a weight of 5500 MU. This plan represented an optimized plan in which the motion of the MLC leaves was taken into account during optimization (CKA4). Finally, interpolated nodes at 1° intervals and 20 apertures between these interpolated nodes were introduced to create plan CKA5.

### 2.5 Beam arrangements and comparison of techniques

**Figure 4:**
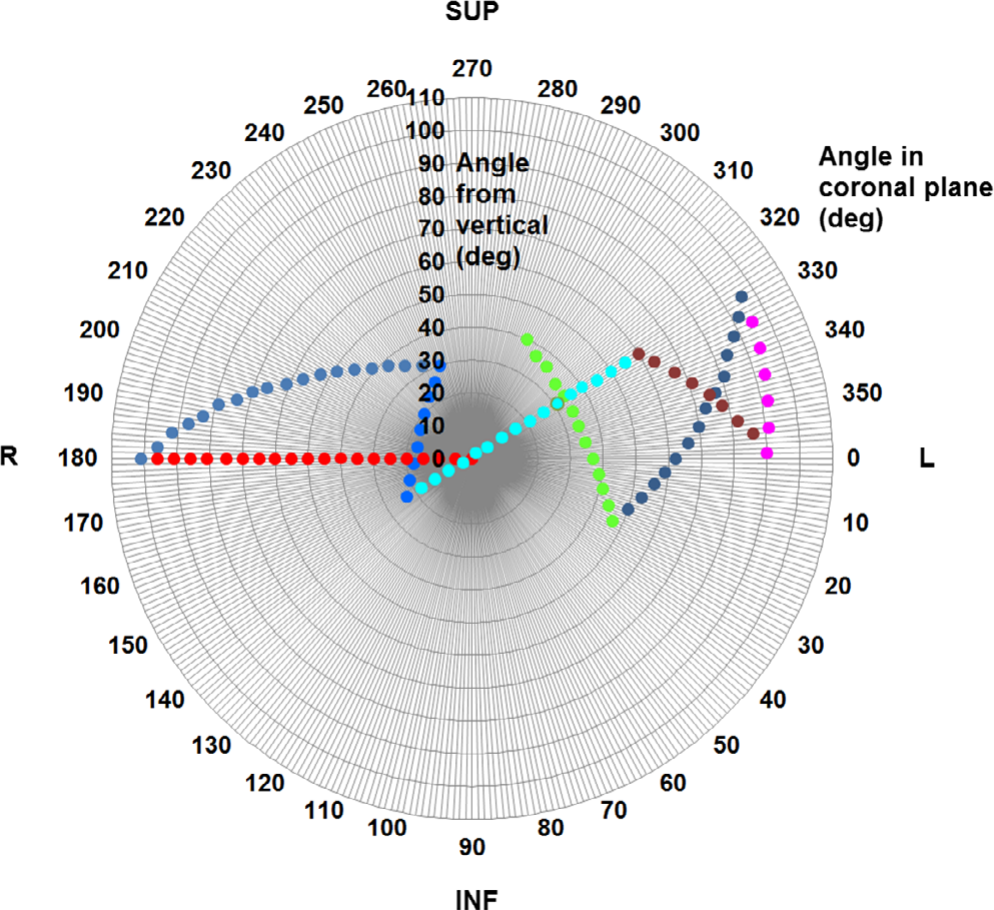
The trajectory used for the Cyberknife Arc robot paths. The diagram views the trajectory looking vertically downward, with increasing distance from the center indicating a more horizontal beam orientation. The patient orientation refers to a patient in the head‐first supine position. Angle from vertical corresponds to gantry angle and angle in coronal plane corresponds to couch angle on a C‐arm linear accelerator (IEC61217 convention). [Color figure can be viewed at http://www.wileyonlinelibrary.com]

In order to avoid the possibility of robot collisions, a fixed arc trajectory was used for all dynamic arc cases. This is illustrated in Fig. 4. The trajectory consisted of eight connected arcs with a total of 104 control points (nodes), spaced at 5° in robot orientation. The trajectory was generated by sampling the standard body path for the Cyberknife so as to obtain a path between existing nodes. The goal was to provide even coverage of the space of orientations, while respecting constraints due to collision avoidance, robot joint limitations, and cable management.

**Figure 5:**
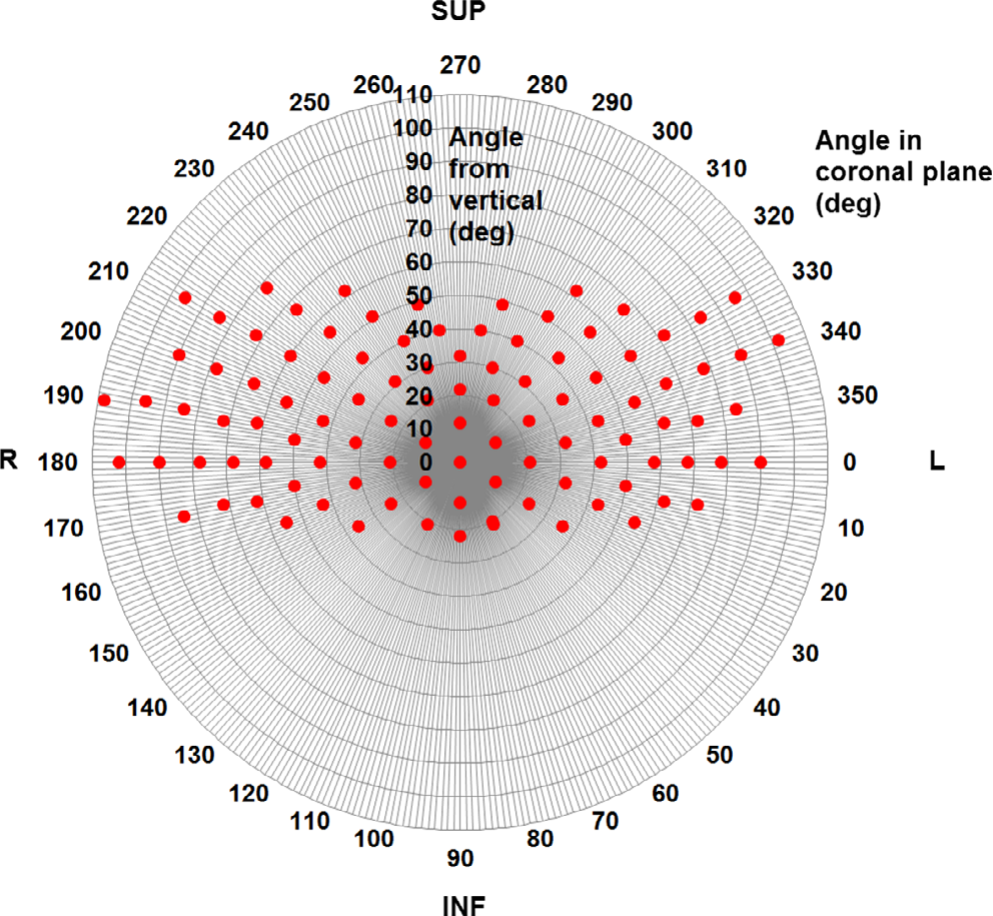
The beam orientations used for the Cyberknife beams with static beam orientations robot paths. The diagram views the orientations looking vertically downward, with increasing distance from the center indicating a more horizontal beam orientation. The patient orientation refers to a patient in the head‐first supine position. Angle from vertical corresponds to gantry angle and angle in coronal plane corresponds to couch angle on a C‐arm linear accelerator (IEC61217 convention). [Color figure can be viewed at http://www.wileyonlinelibrary.com]

The arc trajectory was compared with the standard body path for the Cyberknife. This consisted of 110 nodes, distributed as shown in Fig. 5. The maximum number of apertures allowed by the optimizer for the body path was 110 in order to ensure that any differences in the comparison with the dynamic arc were due to the use of arc delivery, and not simply due to a differing number of apertures. This choice of 110 apertures corresponded approximately to one aperture per node, although the optimizer had the flexibility to use more than one aperture at a single node of the plan and then avoid using an aperture at another node. It was recognized that the number of nodes of the body case (110) was not identical to the number of nodes of the arc plan (104), but these numbers of nodes were considered to be sufficiently close for practical purposes. Both the dynamic arcs and step‐and‐shoot plans used a fixed isocenter, which was located at the center of the planning target volume.

The treatment plans were compared in terms of numbers of segments and numbers of monitor units per fraction. A dosimetric comparison was carried out by comparing dose‐volume histograms and by considering the ability of the methods to meet the clinical goals for each treatment site. The conformity index was also used, and calculated as:

(10) $$CI=\frac{PTV_{\mathrm{pres}}}{\mathrm{PTV}}\times \frac{PTV_{\mathrm{pres}}}{V_{\mathrm{pres}}},$$

where *PTV_pres_* was the volume of the PTV receiving the prescribed dose, *PTV* was the whole PTV volume, and *V_pres_* was the total volume encompassed by the prescribed dose. The first ratio indicated the success of the plan in covering the planning target volume, and the second ratio reflected the avoidance of tissue outside of the planning target volume.^20^ Note that in the case of SBRT, where approximately 95% of the PTV was receiving the prescribed dose, the maximum value expected to be achieved by this conformity index was 0.95.

### 2.6 Treatment time estimation

Treatment times were estimated as follows. For CKA dynamic delivery, treatment time was estimated by considering each node–node interval, assuming that the MU at node *N* were delivered using a constant dose rate as the robot moved between *N* − 1 and *N*:

(a) If MU <= *DT_R_* where *D* was the dose rate in MU/min and *T_R_* was the time taken to traverse the node–node distance with the robot moving at full speed, then it was assumed that this was delivered by moving the robot at full speed while decreasing the linac dose‐rate (or closing the leaves at some point during this motion). In this case the delivery time for this interval was *T_R_*. In this study *T_R_* = 1.5 s.
(b) If MU > *DT_R_*, then the robot had to slow down to deliver this setting, and the delivery time for this interval was MU/*D* mins.

Dynamic delivery times, *t*, in seconds for each node transition were therefore estimated as:

(11) $$t=\max\left(\frac{60}{D}M,\;T_{R}\right),$$

where *M* was the number of monitor units at that node per fraction. *T_R_* = 1.5 s was the robot traversal time.

For CKSB delivery, each segment took 3.5 s for positioning of the MLC, followed by the time taken to deliver the monitor units, based on a dose rate of 1000 MU/min. The robot positioning time for each node was taken to be 1.5 s. This was included in the 3.5 s MLC positioning time as the robot motion and MLC motion occurred simultaneously. However, if no monitor units were delivered at a particular node, the 1.5 s robot positioning time was used and the 3.5 s MLC positioning time was omitted.

### 2.7 Patient cases

Four patient cases were retrospectively investigated in this study: prostate, lung, liver, and left partial breast. The prostate case was planned both for treatment with a homogeneous dose distribution (prostate A), and for treatment with a brachytherapy‐like dose distribution (prostate B). The cases are summarized in Table 3. All treatment plans were for an SBRT technique, with dose to 95% of the PTV being required to receive at least the prescribed dose.

**Table 3:** Summary of cases investigated.

Case	PTV volume (cm^3^)	Prescribed dose (D_95%_) (Gy)	Fractions	Protocol
Prostate A	112.8	36.25	5	RTOG 0938^34^
Prostate B	87.7	38.00	4	Fuller et al.^35^, ^36^
Lung	14.1	50.00	5	RTOG 0813^37^
Liver	27.8	42.75	3	Vautravers‐Dewas et al.^38^
Partial breast	89.5	35.00	5	RTOG 0413^39^

## 3 Results

### 3.1 Illustration of motion between control points

**Figure 6:**
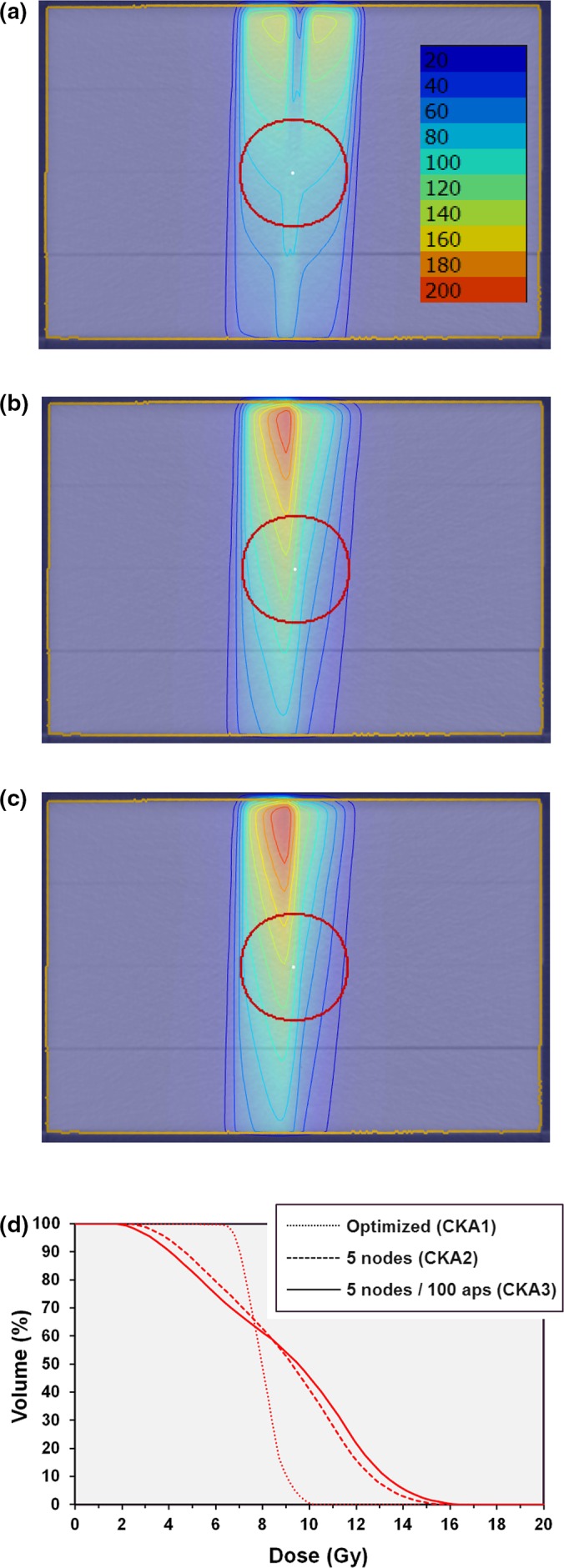
Results of irradiating a spherical planning target volume with two control points. (a) dose calculated at the discrete nodes only (CKA1); (b) five interpolated nodes added between each optimization node (CKA2); (c) five interpolated nodes and 100 interpolated apertures added between each optimization node (CKA3); (d) dose‐volume histograms for the three scenarios. Isodoses are in percentages of 10 Gy. [Color figure can be viewed at http://www.wileyonlinelibrary.com]

The results of the illustrative simulations using two control points are shown in Fig. 6. The two semicircular apertures of Plan CKA1 cover the PTV uniformly when no motion is considered [Fig. 6(a)]. However, when interpolated nodes are included, the dose distribution changes considerably [Fig. 6(b)]. The effect is even greater when additional interpolated apertures are included [Figs. 6(c) and 6(d)].

**Figure 7:**
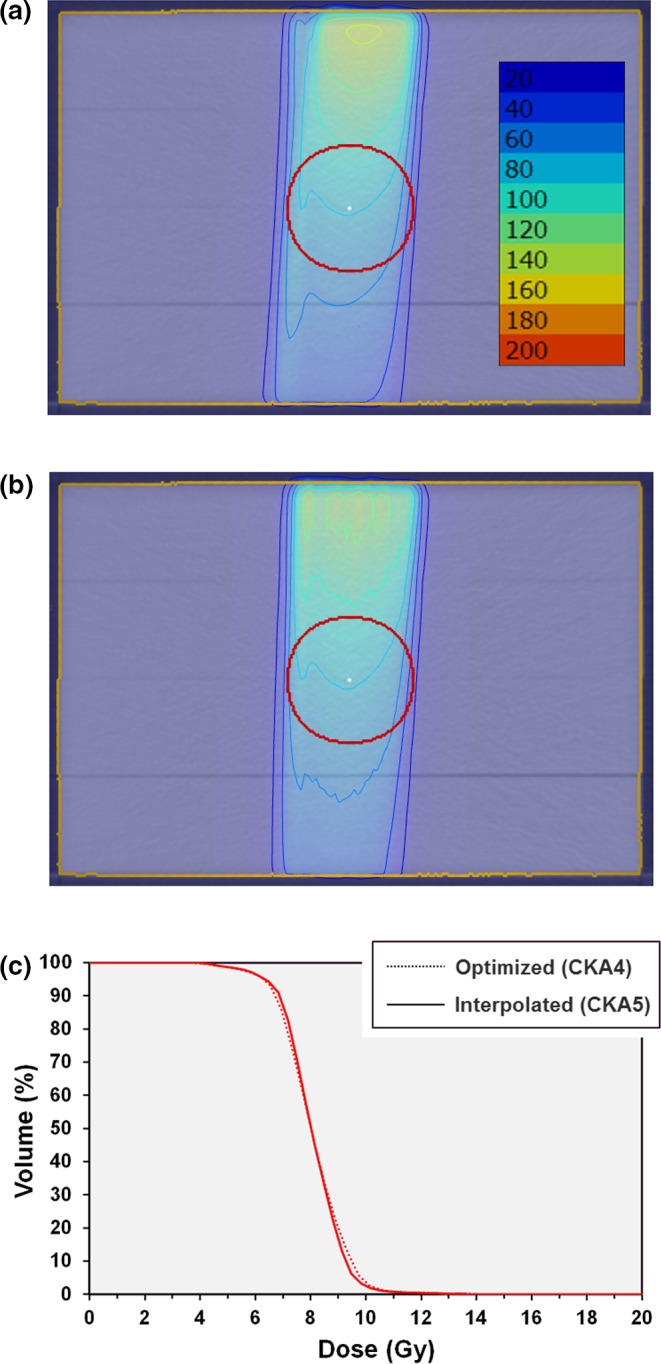
Results of irradiating a spherical planning target volume with two control points. (a) dose calculated continuously between the discrete nodes (CKA4); (b) five interpolated nodes and 100 interpolated apertures added between each optimization node (CKA5); (c) dose‐volume histograms for the two scenarios. Isodoses are in percentages of 10 Gy. [Color figure can be viewed at http://www.wileyonlinelibrary.com]

The second plan, using a narrow aperture and calculated by the continuous method so as to model the motion of the aperture, provides a uniform distribution (Fig. 7). When the plan is recalculated with interpolated nodes and apertures, the dose distribution is almost unchanged, although there are some minor differences in dose distribution superficially.

### 3.2 Comparison of dose calculation methods

**Figure 8:**
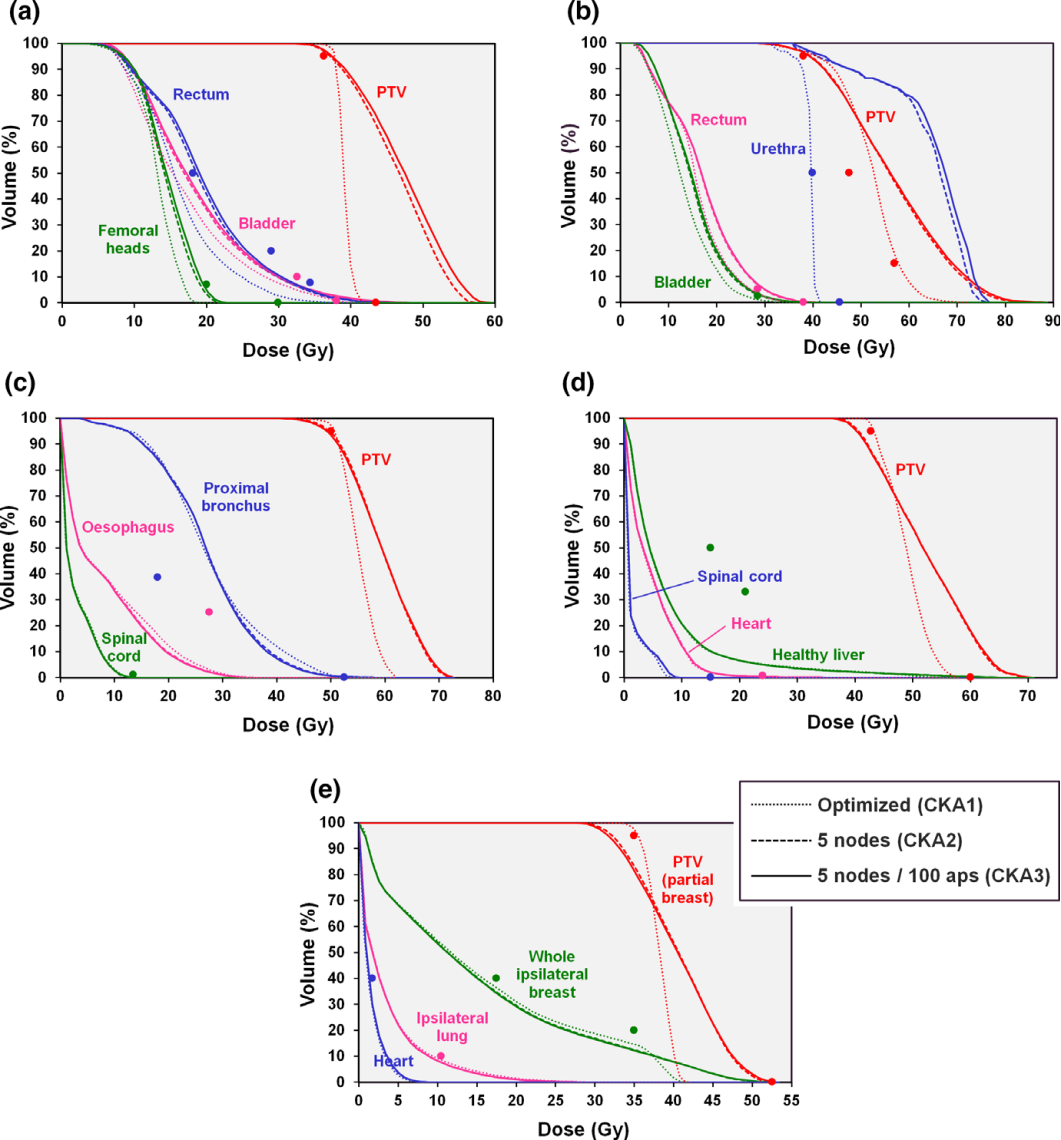
Comparison of dose‐volume histograms for dose calculation based on discrete nodes. Dotted lines: result of optimization based on dose delivered at the discrete nodes only (CKA1); dashed lines: five interpolated nodes added between each optimization node (CKA2); solid lines: five interpolated nodes and 100 interpolated apertures added between each optimization node (CKA3). (a) Prostate A case, (b) prostate B case, (c) lung case, (d) liver case, and (e) partial breast case. The points show the principal clinical constraints for the planning target volume (PTV) and organs at risk. [Color figure can be viewed at http://www.wileyonlinelibrary.com]

The results of CKA optimization without considering dose delivered between the nodes are shown for the patient cases in Fig. 8. The optimized plans meet the clinical constraints but when intermediate nodes are introduced for the recalculation, the dose distribution changes significantly and the clinical goals are no longer met. The effect is even more accentuated when 100 interpolated apertures are included between optimization nodes. Taking this latter case, that is, five interpolated nodes and 100 interpolated apertures, to be the most accurate representation of the true delivered dose, it is clear that the optimization result is not sufficiently accurate.

**Figure 9:**
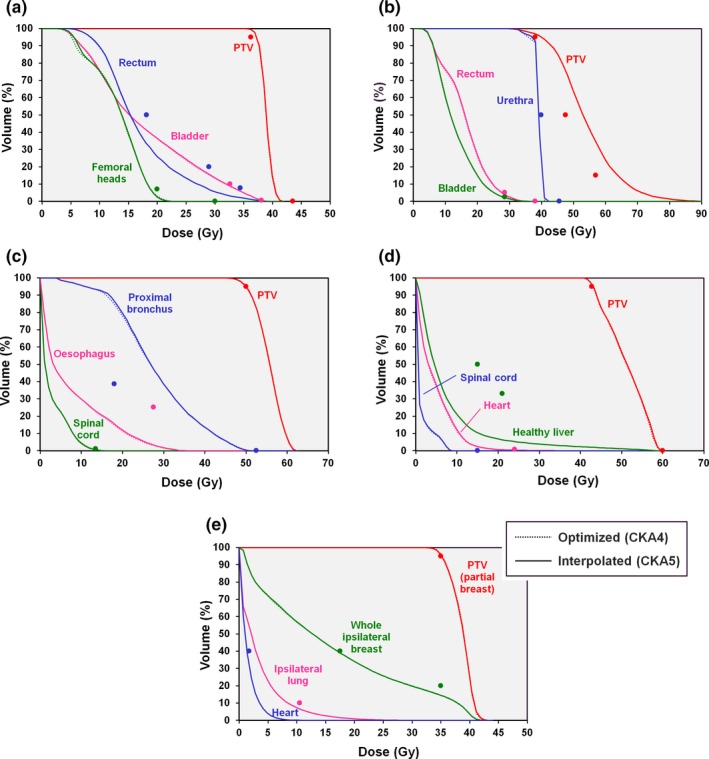
Comparison of dose‐volume histograms for continuous dose calculation as used during optimization and additional node interpolation. Dotted lines: result of optimization based on dose delivered between the discrete nodes using effective fluence (CKA4); solid lines: five interpolated nodes and 100 interpolated apertures added between each optimization node (CKA5). (a) Prostate A case, (b) prostate B case, (c) lung case, (d) liver case, and (e) partial breast case. The points show the principal clinical constraints for the planning target volume (PTV) and organs at risk. [Color figure can be viewed at http://www.wileyonlinelibrary.com]

Instead, it is necessary to include the effect of MLC motion in the optimization itself. Figure 9 shows a comparison of the dose‐volume histograms after optimization using this approach and after final recalculation with five interpolated nodes and 100 interpolated apertures between each optimization node. Again taking the latter to represent the delivered dose distribution, it is clear that the dose calculated by the optimizer, modeling the MLC motion using the method of Christiansen et al.^9^ is accurate. Only the femoral heads (prostate A case), urethra (prostate B case), and proximal bronchus (lung case) show any appreciable divergence in dose between the two calculations, and these changes are very small.

Having established that CKA4, that is, optimization including modeling of MLC motion using effective fluence, provides accurate doses, this approach is used for the subsequent comparison with CKSB.

### 3.3 Comparison of CKA4 and CKSB

Table 4 shows the number of segments used by the CKA4 and CKSB techniques. With CKA4, if one node is not used for dose delivery, the number of segments reduces, due to the nature of the delivery technique. In general, the CKSB technique uses almost all of the allowed segments for delivery of dose. For CKSB, some node positions have two or more segments, while other node positions have zero segments, so that the total allowed number of segments is respected.

**Table 4:** Plan statistics for the five patient cases.

	CKA4 SEGS	CKSB SEGS	CKA4 MU per fraction	CKSB MU per fraction	CKA4 MU per Gy	CKSB MU per Gy
Prostate A	82	108	4978	4231	687	584
Prostate B	84	110	10 579	10 440	1114	1099
Lung	98	110	4312	4402	431	440
Liver	99	107	9368	9496	657	666
Partial breast	79	99	2833	2708	405	387

The monitor units used by CKA4 and CKSB are very similar (Table 4) due to the similar MLC leaf positioning constraints used for both methods. The monitor units per Gy prescribed dose vary according to the complexity of the case, with the prostate B case using the most monitor units per Gy due to the need to spare the urethra within the PTV.

**Figure 10:**
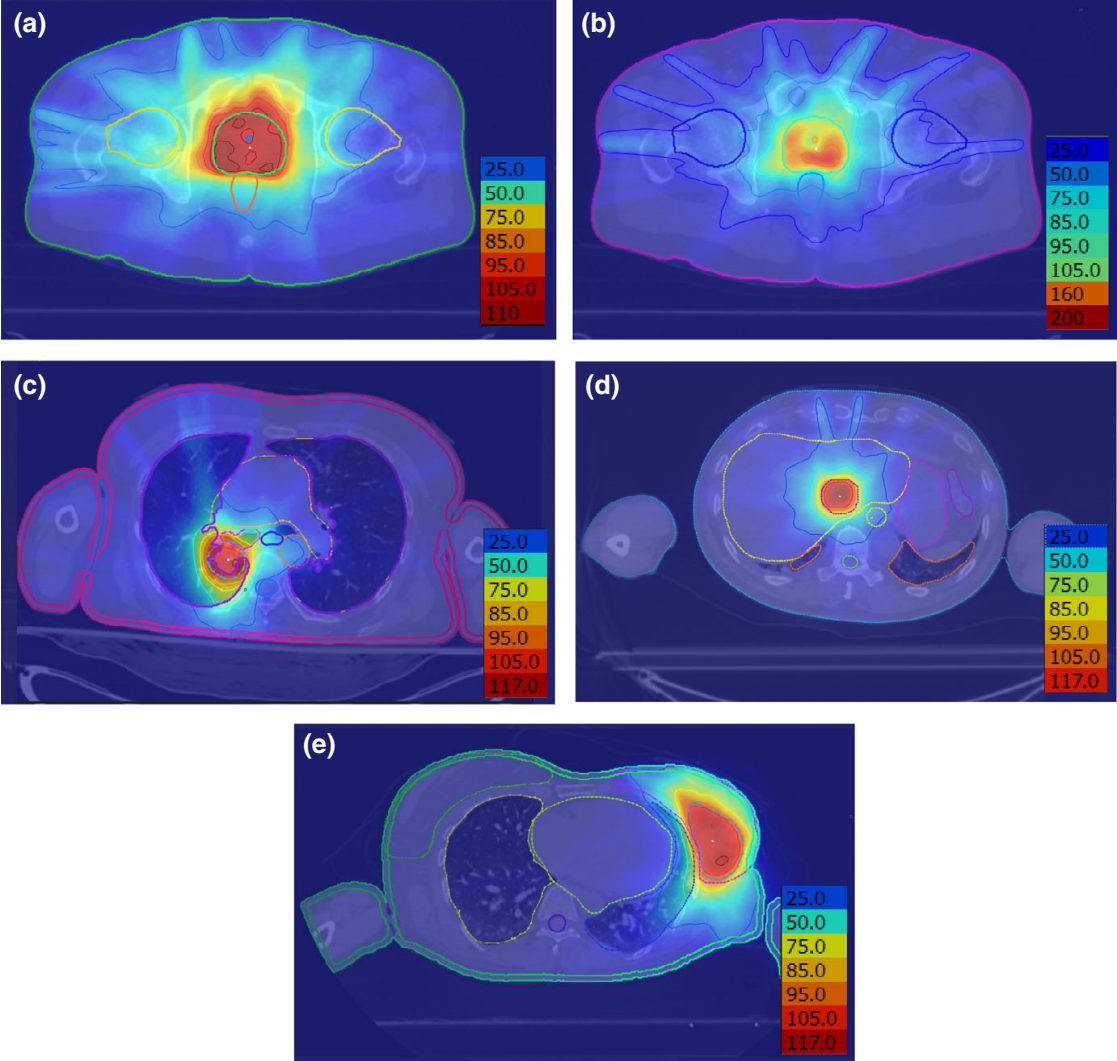
Transaxial dose distributions for the CKA4 plans. The color scheme for the isodoses and colorwash is shown in the bottom right corner of each case, as percentages of the prescribed dose. (a) prostate A, (b) prostate B, (c) lung, (d) liver, (e) partial breast. [Color figure can be viewed at http://www.wileyonlinelibrary.com]

**Figure 11:**
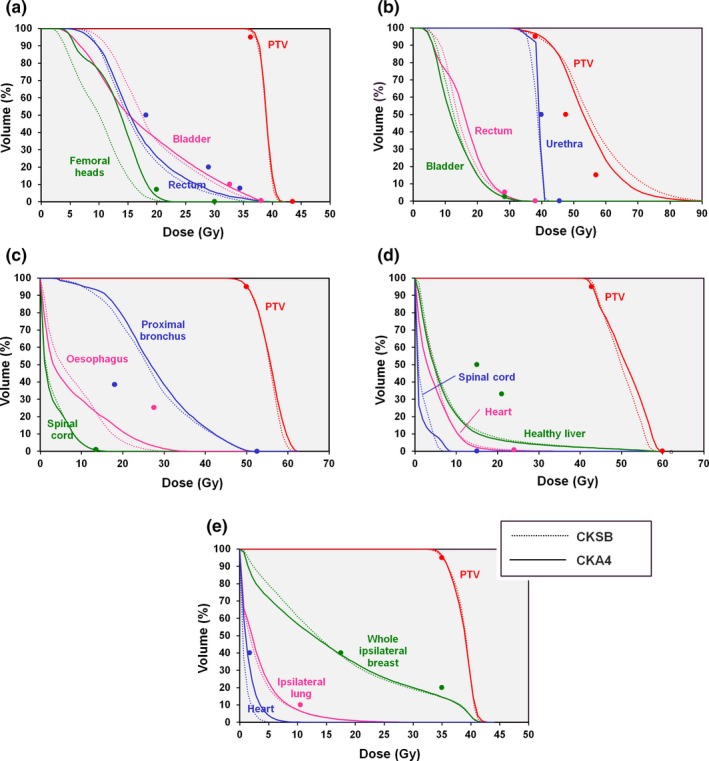
Comparison of dose‐volume histograms for CKA4 and CKSB. (a) Prostate A case, (b) prostate B case, (c) lung case, (d) liver case, and (e) partial breast case. The points show the principal clinical constraints for the planning target volume (PTV) and organs at risk. [Color figure can be viewed at http://www.wileyonlinelibrary.com]

The resulting dose distributions for CKA4 in the five patient cases are shown in Fig. 10. The method is able to provide conformal dose distributions with well‐dispersed peripheral dose. Corresponding dose‐volume histograms comparing CKA4 with CKSB are shown in Fig. 11. In general, the plan quality is comparable between the two techniques, with PTV dose showing no specific trend. The critical structure doses are slightly higher with CKA4, due to the slightly fewer nodes in this plan, and due to the dose delivered between the nodes as the MLC leaves transition from one node to the next. All plans, both CKA4 and CKSB, meet the clinical goals, with the exception of the dose to 4 cm^3^ of proximal bronchial tree in the lung case, where the overlap of the bronchial tree with the PTV means that this statistic reaches approximately 30 Gy, in contrast to the 18 Gy required. This constraint is violated by both the CKA4 and CKSB plans.

The conformity indices in Table 5 show that the dose conformity is comparable for the two methods. Only in the prostate A case is the conformity index substantially lower with CKA4 than with CKSB. This appears to be due to the optimizer selecting certain, mostly anterior, directions as they are beneficial to the avoidance of the critical structures, with the effect that the prescription dose is somewhat spread out.

**Table 5:** Estimated conformity indices and delivery times for the five patient cases.

	CKA4 CI	CKSB CI	CKA4 delivery time (s)	CKSB delivery time (s)
Prostate A	0.80	0.86	355	675
Prostate B	0.64	0.62	672	1025
Lung	0.74	0.71	290	672
Liver	0.95	0.95	584	965
Partial breast	0.93	0.91	235	554

Estimated delivery times are also shown in Table 5. The CKA4 plan is expected to be much faster to deliver than using CKSB, mainly due to the absence of the 3.5 s MLC positioning time between delivery of segments. The median speed improvement factor, taken as a ratio of the treatment delivery times, is 1.90 for the five cases, so in general, the CKA4 method is expected to be about twice as fast as CKSB.

## 4 Discussion

The Cyberknife radiotherapy system has shown itself over the past decade to be a valuable method of delivering a high‐quality treatment, particularly for SBRT.^21^, ^22^, ^23^, ^24^ One of its limitations is the long delivery time. For MLC treatments, this is because robot motion, MLC leaf motion, and beam‐on are all largely performed in a serial fashion. The time taken for imaging in order to track the tumor increases this treatment time further. The possibility of delivering the radiation dynamically using the CKA4 technique is therefore very attractive, as the delivery time without imaging is expected to be reduced by a factor of two.

In order to realize the potential benefit of the dynamic arc, it is necessary to establish a strategy for accurate optimization of the treatment plan. In this study, the modeling of MLC leaf motion has been shown to be important for the accurate calculation of dose in the SBRT plans studied. When using large apertures, for example with VMAT on a C‐arm linear accelerator, it is sufficient to treat the delivered dose as a summation of doses relating to the apertures defined at the control points. So long as the control points are spaced by no more that around 2°, this approach is accurate.^4^ However, when the apertures are small and the MLC is able to move with a considerable speed, the fluence calculated by this method is inadequate to model the actually delivered fluence.^9^ One means of overcoming this inadequacy is to use more closely spaced control points. However, as Kearney et al.^8^ indicate, this leads to a complex search space for the direct aperture optimization, and additionally makes the optimization problem very time‐ and memory‐intensive. Kearney et al.^8^ introduce final control points at 2° spacing.

An alternative method of calculating effective fluence between control points is described by Bedford^19^ and can be used with more coarsely spaced control points to provide an accurate dose during optimization. A mathematical means of performing the same calculation is also described by Christiansen et al.^9^ These methods use the beam orientations of the discrete control points but use the fluence of the moving aperture as it moves between control points. The present study takes the most accurate representation of what is actually delivered to be the optimized plan with segments at 5° intervals recalculated with interpolated segments at 1° intervals and with the method of Christiansen et al.^9^ or Bedford^19^ used between these interpolated segments. When the optimization is based on discrete apertures at 5° node spacing, a significant difference between the optimized dose and the recalculated dose is seen, indicating that the dose during optimization is not accurate. However, when the MLC motion is incorporated into the optimization, very little change occurs when recalculating, demonstrating that the method of modeling leaf motion at the node spacing of 5° is accurate. Similar results are shown by Christiansen et al.^9^ for conventional VMAT treatments.

A limitation of the present study is the use of a dose calculation based on summation of individual bixel doses. This is known to be less accurate than calculating dose based on complete apertures, where the output factors for the apertures are fully taken into account. However, this work uses the bixel dose calculation consistently throughout, so that the dosimetry of the different methods in relation to each other should be accurate. Some simplifications have also been made in the parameters used for modeling the dynamic and static beam deliveries. For example, the beam is not prohibited from delivering low numbers of monitor units, which may cause inaccuracy in ramp up of the beam or difficulty in operating at a low dose rate. However, the numbers of control points where the monitor units per fraction are <5 are only a few percent, and the proportions of the monitor units delivered in such small dose increments are therefore negligible.

Using the accurate method of MLC motion modeling, the exact speed improvement factor for CKA4 with respect to CKSB found in this study is 1.90 (range 1.53 to 2.36), which compares slightly favorably with that of Kearney et al.^8^ They report for prostate and brain patients a speedup of 1.5 ± 0.3, depending on the parameters used by the optimizer. They also use a comparable number of nodes to initialize the arc optimization as is used for the conventional Cyberknife method, so that the comparison of static and dynamic techniques is equal. For circular collimators as opposed to MLC, the same authors also report a speedup of 1.5 to 2.0 for use of an arcing technique.^7^  In the context of a C‐arm linear accelerator, Wild et al.^15^ report a predicted delivery time of 6.5 min on average for noncoplanar VMAT, 1.6 min longer than a coplanar plan, but 2.8 min faster than a noncoplanar intensity‐modulated radiation therapy (IMRT) plan of similar quality. The limiting factor for treatment time is the dose rate of the accelerator. For the large fraction sizes used in the hypofractionated context, a significant time is required to deliver the prescribed number of monitor units.

The parameters chosen to estimate the delivery times in this study are realistic, but some simplifications have been made, compared to the way that the Cyberknife system currently operates. In particular, it is assumed that all delivery nodes are visited during a treatment fraction, whereas in reality, the system minimizes the trajectory taken to visit the nodes used for dose delivery. Consequently, the treatment times for CKSB may be slightly overestimated. On the other hand, for the CKSB path, it is assumed that the robot moves at full speed between nodes, without accelerating or decelerating, taking 1.5 s to make the transition (or 3.5 s if the MLC leaves are repositioned and dose delivered at the new node). However, in reality, the system is known to take longer than this to complete a node transition, so the CKSB delivery time is an underestimation in this respect.

The selection of an appropriate trajectory for CKA4 lends itself to a beam selection algorithm for positioning control points.^3^, ^8^, ^15^, ^25^, ^26^, ^27^ However, the chosen method must include an accurate collision model for the prevention of collisions between the robot and the patient or couch.^26^ Consequently, this study uses a fixed trajectory for all cases. The resulting treatment plans show similar quality to the treatment plans produced using the conventional body path. Comparable results for arcing plans are shown by Kearney et al.^8^ with the use of beam orientation selection before direct aperture optimization and final control point interpolation. The equivalence of arcing and static treatment plans also mirrors the situation with VMAT vs step‐and‐shoot IMRT on conventional linear accelerators.^28^, ^29^, ^30^, ^31^, ^32^, ^33^

For this study, the speed parameters have been chosen based on realistic values for the current Cyberknife hardware and the dose calculation engine is from Accuray. This has allowed the study to be as representative as possible of what might be achievable in practice, but this is an independent study and is not therefore intended to accurately reflect any commercial product. The possibility of using the Cyberknife for dynamic arc delivery is valuable as it offers the prospect of decreasing the long delivery times that are typical at present. This study offers an indication of the plan quality and treatment time that is likely to be achievable for SBRT.

## 5 Conclusions

A control point spacing of 5° in robot angle has been shown to be satisfactory for dynamic arc therapy using the Cyberknife equipped with MLC, provided that the motion of the MLC is modeled between control points using approximate methods to include the influence of intermediate MLC apertures between these points. Taking control point spacing of 1° with MLC motion modeling at intermediate 0.05° resolution to be the reference, plans optimized using 5° angle spacing are shown to be accurate to within around 1% in general. Dynamic delivery of Cyberknife treatment provides a dose distribution which is comparable to that created using a static delivery path, for a comparable number of segments. This has been demonstrated for SBRT plans in several different tumor sites. The delivery speed improvement when using such a dynamic treatment is around a factor of two.

## Conflict Of Interest

This work has been funded by Accuray Inc.
